# Antenatal corticosteroids may contribute to illness in children in the future

**DOI:** 10.20945/2359-3997000000434

**Published:** 2022-01-01

**Authors:** Luiz Augusto Casulari, Lucilia Casulari da Motta

**Affiliations:** 1 Universidade de Brasília Brasília DF Brasil Pós-graduação em Ciências da Saúde, Universidade de Brasília, Brasília, DF, Brasil; 2 Universidade de Brasília Brasília DF Brasil Ginecologia e Obstetrícia, Universidade de Brasília, Brasília, DF, Brasil

**DEAR EDITOR,**

Regarding recommendations for prenatal care for pregnant women at high risk of premature birth, the use of high betamethasone or dexamethasone doses has been recommended. The benefits to the fetus shortly after delivery are well defined, with reduction in neonatal mortality even in underdeveloped countries, without increasing maternal infection rates (1). However, there is increasing evidence that high doses of these glucocorticoids can cause deleterious effects on the health of infants at other stages of life. The beneficial effects of the hypothalamus-pituitary-adrenal axis on homeostasis of the fetus-mother interaction are well known but are not the scope of this letter.

In a large Finnish population study (2), 670,097 pregnant women were evaluated, of which 14,868 (2.2%) used betamethasone (12 mg, every 24 hours for two days). Children were followed up for the first 10 years of life, with follow-up median of 5.8 and interquartile from 3.1 to 8.7 years. The primary outcome of any mental and behavioral disorders were found an entire cohort and term infants, but not in preterm infants. In the evaluations of the secondary outcome, sleep disorders were observed in the entire cohort, term, and preterm infants. Mild, moderate, unspecified intellectual disability were found in entire cohort and preterm infants. Entire cohort and term infants were described with: psychological development disorders; attention-deficit/hyperactivity or conduct disorders; mixed disorders of conduct and emotions; emotional, social functions, or tic disorders; other behavioral and emotional disorders. However, these were not observed in any of the groups: severe, profound intellectual disability; autism spectrum disorders; psychotic, mood, neurotic, stress-related, or somatization disorders; eating disorders. When analyzing the cohort with the Kaplan-Meier curve, it was observed that, over time, after birth until the age of nine years, there was progressive increase in the probability of any diagnosis of mental and behavioral disorders in full-term infants but not in preterm ones.

In another recent publication (3), describes experiments in mice, which show that the time of day when corticosteroids are used predicts the behavioral phenotype in adulthood. If the steroid was administered outside the circadian rhythm phase, cubs showed high anxiety, impaired coping with stress, and dysfunctional regulation of the axis to stress. They have shown that this is due to the control of glucocorticoid receptors in the hypothalamus. In this study (3), carried out in three health centers in Germany, the authors retrospectively analyzed 5-year-old children, whose mothers received the glucocorticoid between 4 am and 12 pm or at 8 pm. Those in which the glucocorticoid was administered in the evening had worse ability to compensate for stress. These results offer insights into the circadian physiology of maternal fetal interference and attribute a role to the fetal clock as a temporal guardian of cortisol sensitivity.

These authors show three implications from their results: 1) the extension of the use of corticosteroids after 34 weeks, as supported in most recent recommendations, can increase the risk of children exposed to corticosteroids; 2) the use of corticosteroids beyond 34 weeks also reduces the risk of respiratory problems, but these are transient and treatable, but can cause long-term problems in these children; 3) 45.27% of those who used corticosteroids before 34 weeks had full-term delivery and the use of corticosteroids would not be necessary.

In an excellent review (4), the authors showed that the use of glucocorticoids during pregnancy has short-term benefits for the fetus, but, throughout life, they can have deleterious consequences in various systems of the body. In experimental animals, exposure to glucocorticoids during pregnancy can lead to central and peripheral changes in the levels of ACTH, cortisol, growth hormone, IGF-1, TSH, thyroid hormones, FSH and LH, gonadal hormones and pancreatic hormones. These important changes can compromise responses to stress, with the development of psychological diseases, metabolic diseases and reproductive dysfunction. Many of the article's conclusions are based on the authors’ research.

The results of cardiometabolic changes in children whose mothers used betamethasone before delivery are not very clear. Small increase in blood pressure, slight decrease in renal clearance and beta-cell function, and evidence of increased aortic stiffness have been described in some studies. However, in others researches antenatal exposure was not associated with more alterations in these children's blood pressure, insulin sensibility, cortisol concentrations, peripheral arterial function, blood lipids and adiposity (see “5” for review).

It was described that neonatal treatment of prematurely born children with dexamethasone, but not with hydrocortisone, resulted in long-lasting programming effects on hypothalamus-pituitary-adrenal axis and on the T-helper 1/T-helper 2 cytokine balance (6).

The results from studies by Nina Alexander and colleagues in the long-term follow-up of German children who received dexamethasone or betamethasone during pregnancy are very interesting. In these children, assessments extended up to the age of 18 years. A total of 209 children aged 6-11 years were studied, all born at full-term to avoid the confounding factor of prematurity. The Acute Psychosocial Stress Test (Trier Social Stress Test for Children) showed significantly higher increase in cortisol levels in children whose mothers received corticosteroids. This change in the hypothalamic-pituitary-adrenal axis was greater in girls than in boys (7). However, in the assessment of cognitive deficit by Cattell's Culture Fair Test in these children at six and eleven years of age, the deleterious effect of previous use of glucocorticoids was not observed (8).

In 2019, (9) evaluated the same sample that had been previously examined (7,8), but already in adolescence, in the age group of 14-18 years. Using the same Trier Social Stress Test, they showed increased cortisol reactivity from childhood to adolescence in the group that used corticosteroids in relation to control. This suggests that the effect of the use of antenatal corticosteroids can persist until adolescence and increase vulnerability to develop stress-related changes.

Other investigations were carried out in the group of children described in previous publications (7,8). Ilg and cols. (10) studied children aged 14-18 years with average age of 16 years. They investigated brain function correlated to the monitoring of cognitive conflicts. They used EEG to show changes in specific brain locations during the modified test of the Cued Continuous Performance Task version.

During the performance of an experimental task that requires conflict monitoring and response inhibition, activations in the parieto-frontal network underlying cognitive flexibility and behavioral control were reduced in full-term adolescents exposed to prenatal glucorticoid. Individual differences in recruitment in the anterior cingulate and pre-cuneiform network are, respectively, associated with variations in the response consistency and reactivity of cortisol to stress.

Taken together, the authors argue that the prenatal use of corticosteroids has lasting impacts on the development of parieto-frontal functions of the brain during adolescence, affecting multiple factors of adaptive cognition and behavioral control.

It is very relevant that there is an increasing number of evidences indicating that glucocorticoid programming effects may not be limited to the first directly exposed individuals but may be transmissible across generations; for instance, alterations in glucocorticoid-induced DNA methylation have been observed up to the second generation following hormonal exposure (11).

The purpose of this letter, when describing the results of recent and important research, is to suggest a review of current recommendations regarding the use of high doses of synthetic glucocorticoid in pregnancies of high risk of premature birth.
